# Extruded PLA Nanocomposites Modified by Graphene Oxide and Ionic Liquid

**DOI:** 10.3390/polym13040655

**Published:** 2021-02-22

**Authors:** Cristian Sánchez-Rodríguez, María-Dolores Avilés, Ramón Pamies, Francisco-José Carrión-Vilches, José Sanes, María-Dolores Bermúdez

**Affiliations:** Grupo de Ciencia de Materiales e Ingeniería Metalúrgica, Universidad Politécnica de Cartagena, Campus de la Muralla del Mar., 30202 Cartagena, Spain; cristian.sanchez@upct.es (C.S.-R.); mdolores.aviles@upct.es (M.-D.A.); fjc.vilches@upct.es (F.-J.C.-V.); pepe.sanes@upct.es (J.S.); mdolores.bermudez@upct.es (M.-D.B.)

**Keywords:** PLA nanocomposites, extrusion, DMA, Raman, FT-IR, graphene, ionic liquids

## Abstract

Polylactic acid (PLA)-based nanocomposites were prepared by twin-screw extrusion. Graphene oxide (GO) and an ionic liquid (IL) were used as additives separately and simultaneously. The characterization of the samples was carried out by means of Fourier transform infrared (FT-IR) and Raman spectroscopies, thermogravimetric analysis (TGA), and differential scanning calorimetry (DSC). The viscoelastic behavior was determined using dynamic mechanical analysis (DMA) and rheological measurements. IL acted as internal lubricant increasing the mobility of PLA chains in the solid and rubbery states; however, the effect was less dominant when the composites were melted. When GO and IL were included, the viscosity of the nanocomposites at high temperatures presented a quasi-Newtonian behavior and, therefore, the processability of PLA was highly improved.

## 1. Introduction

Despite the increasing number of plastic appliances which are reused or recycled, massive quantities of plastic are accumulated in landfills. This final disposal option is causing major problems in the environment because plastics accumulate in seas and oceans. Some decades ago, biodegradable materials started to attract the attention of the scientific community in order to reduce the environmental impact of domestic plastics [1,2], and more recently these materials have been replacing traditional plastics [3,4]. Polylactic acid (PLA) is an aliphatic polyester which is synthesized by condensation of lactic acid. This material is a bio-based thermoplastic which is biodegradable and biocompatible due to its low toxicity [5,6,7]. It has been extensively used in biomedical applications [8,9], protection of crops [10], and food packaging [11,12]. In general, the mechanical properties of PLA are similar to those of common fossil fuel-based thermoplastics; however, the glass transition temperature (T_g_), ductility, and thermal stability are lower. Thus, different types of additives are commonly used to improve the properties of PLA [13,14,15,16].

Ionic liquids (ILs) are molten salts under 100 °C [17] containing organic cations and anions which can be either organic or inorganic. These salts present low volatility and flammability, and high thermal stability. Their unique properties have promoted different applications such as, solvents for organic reactions, tailored lubricants [18,19], high-performance liquid electrolytes [20], and plasticizers and additives in polymer-based composite materials [21,22,23].

Inorganic nanofillers (size < 100 nm) are widely used to enhance the thermal, mechanical, and barrier properties of thermoplastics. In the case of PLA, nanosilicates, ZnO nanoparticles, nanoclays, and carbon nanophases are commonly used, among others [24,25,26,27]. Recently, the development of graphene-based nanocomposites has experienced a growth due to the improvement of their properties, especially when graphene oxide (GO) is utilized [28,29,30]. GO is a graphenic nanophase with oxygen containing functional groups on the surface and an interlayer distance of 8.4 Å [31]. The presence of hydroxylic, carboxylic, carbonylic, and epoxy groups on the surface of GO enables an easy functionalization of the nanophase [32,33,34]. The addition of graphene-derived nanophases and ILs on PLA has shown a more homogeneous dispersion in the matrix [35,36].

Melting processing of nanocomposites is a versatile and efficient manufacturing process which can be rapidly applicable of industrial scale [37]. In the case of PLA-based nanocomposites, twin-screw extrusion is widely used since the mixing action is based on elongation flow [38,39,40,41]. In this work, we have explored the effects of the addition of GO and/or an IL on a polymeric matrix of PLA in order to optimize processing conditions and improve the properties of the PLA-based nanocomposites. The ionic liquid 1-Butyl-1-Methylpyrrolidinium Hexafluorophosphate ([Bmpyr] PF6) was selected due to its solid state at room temperature, simplifying the addition to the plastic. Although this IL has been used previously in elastomeric blends [42], it is the first time that it is added to a thermoplastic matrix, to the best of our knowledge. The nanocomposites were prepared by twin-screw extrusion and the characterization of the samples was carried out by means of Fourier transform infrared (FT-IR) and Raman spectroscopy. Thermogravimetric analysis (TGA) and differential scanning calorimetry (DSC) tests were performed to determine the thermal properties. The viscoelastic behavior was explored using dynamic mechanical analysis (DMA) and rheological techniques.

## 2. Materials and Methods

### 2.1. Materials

**Figure 1:**
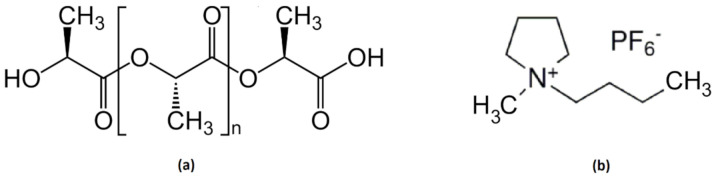
Chemical structure of Poly-lactic acid (PLA) (**a**) and ionic liquid (IL) (**b**).

PLA powder with reference RXP 7503 NATURAL (PLA/9/1000μ) was manufactured by Natureworks (Blair, NE, USA) and supplied by Resinex Group (Tarragona, Spain). The tradename of this polymer is IngeoTM biopolymer and the chemical structure is depicted in Figure 1a. The average molecular weight of this batch is 144,000 g/mol and has a melting point of 175 °C. The ionic liquid, 1-Butyl-1-Methylpyrrolidinium Hexafluorophosphate with CAS number (330671-29-9), was supplied by Solvionic SA (Toulouse, France) as a white powder at room temperature with a purity of 99% and a melting point of 85 °C. The chemical structure is shown in Figure 1b. Graphene oxide with code av-GOx-40 (lot G401218) was supplied by Avanzare Innovación Tecnológica S.L. (La Rioja, Spain), with the technical information shown in Table 1.

### 2.2. Samples Preparation

Samples were prepared by mechanical blending of dried PLA with the additives to obtain PLA + GO (PGO), PLA + ionic liquid (PLP), or both PLA + GO + IL (PGL). The solid additives were stirred for 5 min. Due to their hygroscopicity, all PLA samples were dried under vacuum for 24 h at 60 °C. The resulting powder was extruded in a co-rotating twin-screw extruder TwinLab 10 from TwinTech Extrusion LTd (Stoke-on-Trent, UK). The processing conditions are detailed in Section 3.1. The extruded filament was pelletized with a roller speed of 80 rpm and a cutter speed of 65 rpm. The concentration of the additives varied from 1 to 5 wt.% in the case of IL, and 0.5 to 1 wt.% in the case of GO. These ranges were chosen after a bibliographic search and an earlier work [22,43,44]. Previous testing showed that the best sample preparation and processability were found when the samples with the concentrations shown in Table 2 were used. These samples were selected for further characterization and study.

### 2.3. Characterization Methods

Two different kinds of thermogravimetric methods were performed with a TGA 1HT equipment, provided by Mettler-Toledo (L’Hospitalet de Llobregat, Spain). Firstly, temperature scans with a heating rate of 10 °C/min from room temperature to 800 °C were carried out to evaluate the decomposition temperature in an inert atmosphere. Secondly, the effect of time on the thermal degradation was studied at a constant temperature set at 170 °C. Differential scanning calorimetry (DSC) tests were carried out with a DSC 882E from Mettler-Toledo (L’Hospitalet de Llobregat, Spain). The samples were heated and cooled at a speed rate of 10 °C/min in dynamic inert atmosphere (50 mL/min). A Fourier transform infrared (FT-IR) spectrometer Thermo Nicolet 5700 was used to characterize the structure of the materials. A 514 nm laser using a Renishaw inVia and a Leica microscope were used to record the Raman spectra, with an integration time of 0.5 s to avoid the sample degradation. This equipment was provided by Witek (Ulm, Germany). The viscoelastic behavior was evaluated by means of a Q800 dynamic mechanical analyzer from TA Instruments (New Castle, DE, USA). The experiment was carried out at an amplitude of 15 µm and a frequency of 1 Hz, with varying temperatures from 35 to 75 °C with a heating rate of 3 °C/min. We have run four experiments per sample. Two different types of rheological tests were conducted. Firstly, the linear viscoelastic region was determined from strain-sweep tests with a constant frequency of 1 Hz. Afterwards, frequency-sweep tests were performed at the constant strain calculated in the previous experiment. Data were recorded at 110, 130, 150, and 170 °C and the presented results are the average of three experiments.

## 3. Results

### 3.1. Processing and Characterization of Pellets

#### 3.1.1. Optimization of the Extrusion of PLA and Its Derivatives

In order to achieve the optimal conditions for the melting processing of PLA, different temperature profiles, high and low, were evaluated [44]. The detailed information of these profiles can be seen in Table 3.

Specific mechanical energy (SME) shows how much energy went into the polymer during the extrusion. High temperature values favor the extrusion since the viscosity of the melted PLA was lower. Therefore, when the temperature was raised, the values of SME were lower. The evaluation of SME was carried out using Equation (1), where *E* is the velocity of the extruder (rpm), *T* is the value of the torque (MJ·m), and *V* is the flow of the material (kg/h), which was calculated according to Equation (2):

(1) SME=(2×π×E×T)/(1000×60×V)

(2) V=3.6×(0.003×F−0.003)

where *F* is the velocity of the feeder (rpm).

We chose four values of *F* (5, 10, 15, and 20 rpm) and four values of *E* (50, 100, 150, and 200 rpm) to evaluate the different operation options. Therefore, 16 combinations of processing conditions were evaluated for each temperature profile. Values of *F* lower than 10 rpm generates filaments with an excessive thin diameter. When *F* was higher than 15 rpm, obstruction problems in the feeder were found. Taking into account the extrusion speed, it was found that the optimum thickness of the filament occurred when *E* was in the range of 100 to 150 rpm. Then a value of 130 rpm was selected. Summarizing, we have collected the optimum values of the extrusion processes of pure PLA in Table 4 for both temperature profiles.

As expected, the SME values were higher in the case of the low-temperature profile since the fluidity of the PLA melt was lower. It was found that higher values of SME improve the interaction between the different components of composites [45], and PLA is a temperature sensitive polymer. Therefore, we selected the low temperature profile as the most suitable conditions to obtain the PLA-based compounds. When these conditions were applied to the processing of PLP, PGO, and PGL, a drop in the values of SME was observed. The addition of GO has a low impact in the value of SME, but when the IL was added there was a reduction of 70%, as it is observed in Table 5.

#### 3.1.2. RAMAN and FT-IR

**Figure 2:**
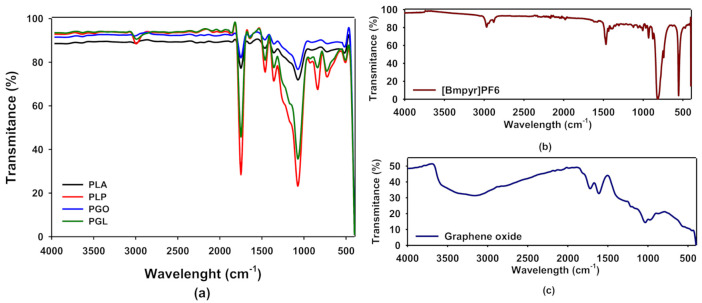
Fourier transform infrared (FT-IR) spectra of PLA, PLP, PGO, and PGL (**a**); [Bmpyr]PF6 (**b**); and graphene oxide (**c**).

In Figure 2a we show the FT-IR spectra of PLA, PLP, PGO, and PGL. The spectra for [Bmpyr] PF6 and GO are shown in Figure 2b,c, respectively. In Table 6, we have collected the typical FT-IR peaks for pure PLA, [Bmpyr] PF6, and GO and their assignments [46,47,48,49]. When the spectra of the composites were examined, the PLA peaks were dominant. In the case of PLP and PGL, we observed the peaks at 814, 871, 2877, and 2964 cm^−1^ which are characteristic of the ionic liquid [50,51,52,53,54]. However, in PGO and PGL the peaks of graphene oxide were not observed.

**Figure 3:**
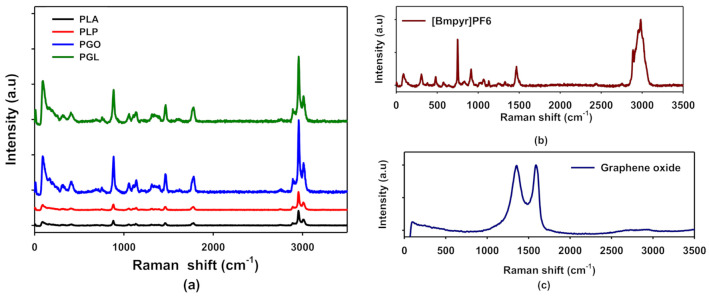
Raman spectra of PLA, PLP, PGO, and PGL (**a**); [Bmpyr]PF6 (**b**); and graphene oxide (**c**).

In Figure 3a, the Raman spectra of PLA, PLP, PGO, and PGL are shown. These results are in agreement with the FT-IR spectra. For PLA, we assigned the peaks at 711, 760, and 1773 cm^−1^ to the C=O bonding, 1179 cm^−1^ to the asymmetric mode of C–O, and the symmetric mode of C–O–C to the peak at 1092 cm^−1^. The peak at 1450 cm^−1^ was assigned to asymmetric C–H and the symmetric CH_3_ modes to 1128 and 1386 cm^−1^ [59]. The pure ionic liquid spectra are shown in Figure 3b. The anion shows two characteristic peaks at 471 and 743 cm^−1^, and the cation presents the stretching of groups CH_3_ at 1462 and 2984 cm^−1^. We also observed an additional peak at 307 cm^−1^ assigned to CH_3_–N–CH which was not seen by means of FT-IR [60]. In the case of graphene oxide (see Figure 3c), we found the 2D band at 2690 cm^−1^, and D and G bands at 1350 and 1585 cm^−1^, respectively [61]. The examination of the composites by Raman was complicated since the concentrations of additives were low, and the peaks of IL were not easily found in samples PLP and PGL. However, the characteristic peak of GO at 1585 cm^−1^ was observed proving that this additive was incorporated to the polymeric matrix in samples PGO and PGL. All these data are collected in Table 7.

#### 3.1.3. Thermal Analysis: TGA and DSC

**Figure 4:**
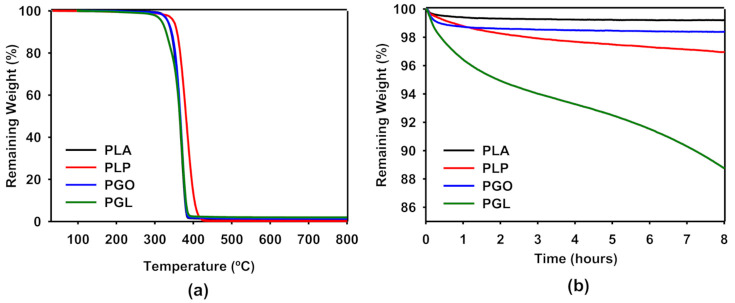
Thermogravimetric analysis of PLA, PLP, PGO, and PG. Effect of temperature on the weight loss from 25 to 800 °C (**a**) and effect of time at a constant temperature of 170 °C (**b**). The average of three experiments is represented.

In Figure 4a, we have shown the thermogravimetric analysis of PLA, PLP, PGO, and PGL. It can be seen that the samples containing graphene (pristine and modified) had an identical behavior compared to PLA, and the three curves overlapped. In the case of the addition of ionic liquid, a shift of the curve was seen to higher temperatures. For all the samples, only one sharp step was found. In Table 8, we have collected the values of degradation temperatures for 5, 50, and 95% of weight loss. It is clear that the addition of [Bmpyr] PF6 enhanced the thermal stability of PLA. The evaluation of the thermal stability with time is shown in Figure 4b. In this study, we set a constant temperature of 170 °C and observed the weight loss over 8 h. The addition of GO and IL did not significantly affect the weight loss in PLA. However, we observed a higher effect in the PGL sample with 11% weight loss after 8 h. This information was useful to design the rheological tests, since the time of the experiment can affect the results due to degradation of the sample.

**Figure 5:**
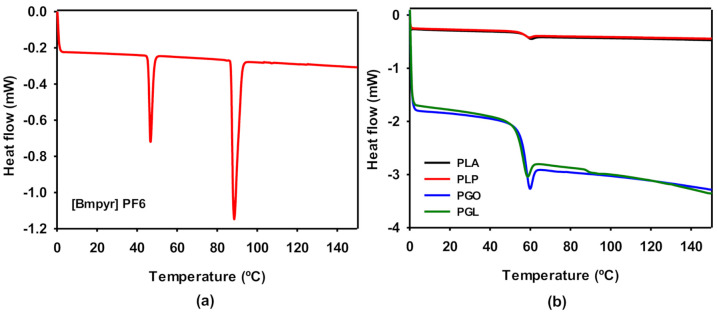
Differential scanning calorimetry (DSC) curves for [Bmpyr] PF6 (**a**) and for PLA, PLP, PGO, and PGL (**b**).

DSC experiments were carried out for pure IL (Figure 5a) and the polymeric samples (Figure 5b). The ionic liquid presented two phase transitions. As it is described above, IL is solid at room temperature. Therefore, the first peak was found at 46 °C, which can be ascribed to the transition from the solid state to liquid crystal. The melting point was found at 88.5 °C, in agreement with the information provided by the supplier. The thermodynamic properties T_g_ and relaxation enthalpy (ΔH_rel_) of PLA, PLP, PGO, and PGL were extracted from the results shown in Figure 5b and collected in Table 9. The different additives did not show a high impact on the glass transition of PLA. However, the effect of the addition of graphene provoked a dramatic decrease in ΔH_rel_. The relaxation enthalpy corresponds to the release upon heating of degrees of freedom after the material had been aged below the T_g_, and therefore it is also related to the mobility of the polymeric chain and the crystallinity of the plastic [61].

### 3.2. Viscoelastic Behavior

#### 3.2.1. Dynamic Mechanical Analysis (DMA)

**Figure 6:**
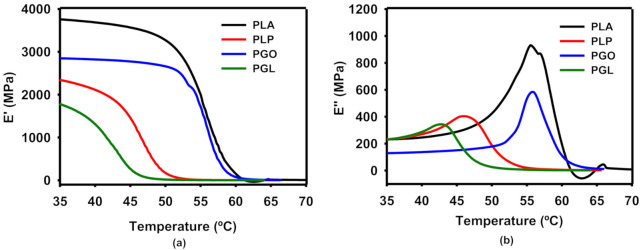
Dynamic mechanical analysis of the effect of temperature on storage (**a**) and loss (**b**) moduli.

The progression with increased temperature of the storage (E’) and loss (E’’) moduli are shown in Figure 6a,b, respectively. The values found for neat PLA are in agreement with the literature in the transition from the glassy to the rubbery regions [62]. The presence of IL in the PLP and PGL nanocomposites led to a lower T_g_, taking into consideration the onset E’ and the peak of E’’, and tan δ methods. In the case of PGL, the T_g_ value of 42.2 °C (E’) was particularly notable in comparison with 55.1 °C (E’) for the neat PLA. These values are shown in Table 10, and the trend was similar despite the method used for the calculation. The addition of GO had a minimum impact on the T_g_ values. However, a drop can be seen for both PLP and PGL, similar to a plasticizer effect [63].

#### 3.2.2. Rheology

**Figure 7:**
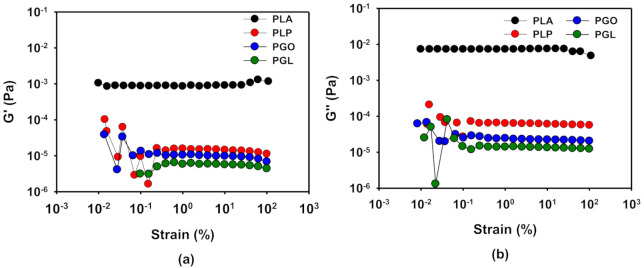
Determination of linear viscoelastic region (LVR) by the study of G’ (**a**) and G’’ (**b**) with strain for the PLA, PLP, PGO, and PGL at 170 °C.

The linear viscoelastic region (LVR) was determined by means of oscillatory experiments with constant frequency of 1 Hz and variable amplitude, as it is described above. The results of PLA at 170 °C are shown as an example in Figure 7, and the results for the four samples at different temperatures can be found in Table 11. As it can be seen, the viscoelastic properties remained stable in a wide range of strain. From these results, a value of 1% of strain was set to perform the frequency sweep tests to investigate the rheological behavior of the samples.

**Figure 8:**
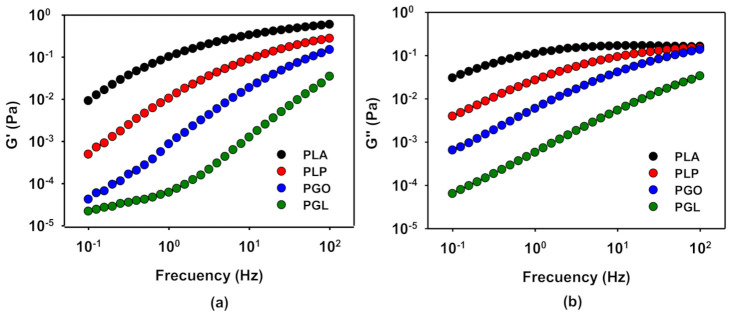
Representation of the viscoelastic properties, G’ (**a**) and G’’ (**b**), in a sweep frequency test at 130 °C for PLA and its GO, IL, and IL-modified GO derivatives.

In order to evaluate the viscoelastic behavior of the samples, frequency sweep tests were carried out at 110, 130, 150, and 170 °C at a constant strain of 1% calculated in the previous section. The time of the experiments was set lower than 20 min to avoid the degradation of the samples at high temperatures, especially sample PGL. G’ and G’’ at 130 °C are represented in Figure 8a,b, respectively. As it can be seen, the viscoelastic properties decreased with the addition of the different additives, in contrast with previous results [64], probably because for this combination of GO and PLA the percolation concentration is not yield [65]. The addition of IL and GO (sample PGL) shows the strongest effect on both G’ and G’’. On the other hand, when only IL was added, the viscoelastic properties of PLA are less affected. In all cases, at low frequencies the storage modulus was more dominant, but with increasing frequency the loss modulus increased until a cross-over point, except for the sample PGL.

**Figure 9:**
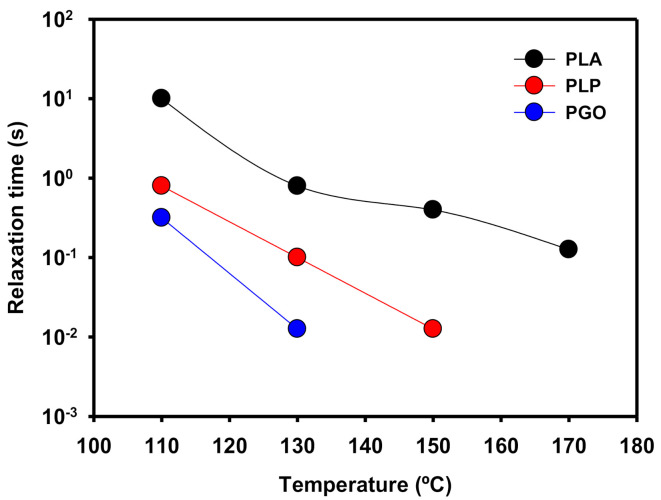
Representation of the relaxation time of PLA, PLP, and PGO at different temperatures.

In Figure 9, the relaxation times (λ) of the samples are shown. These values were calculated as the inverse of the cross-over values of G’ and G’’ when the frequency was increased. When the temperature was raised, the values of λ decreased because the polymeric matrix was able to relax to the steady state in shorter times due to the higher mobility of the molecules. In the case of PLP at 170 °C, there was no crossing point between G’ and G’’ because the loss modulus was dominant in all the range of the studied frequencies. In the case of the addition of GO, the same behavior was found at temperatures higher than 150 °C. However, when both additives were included the viscous modulus was higher than the elastic modulus even at 110 °C.

**Figure 10:**
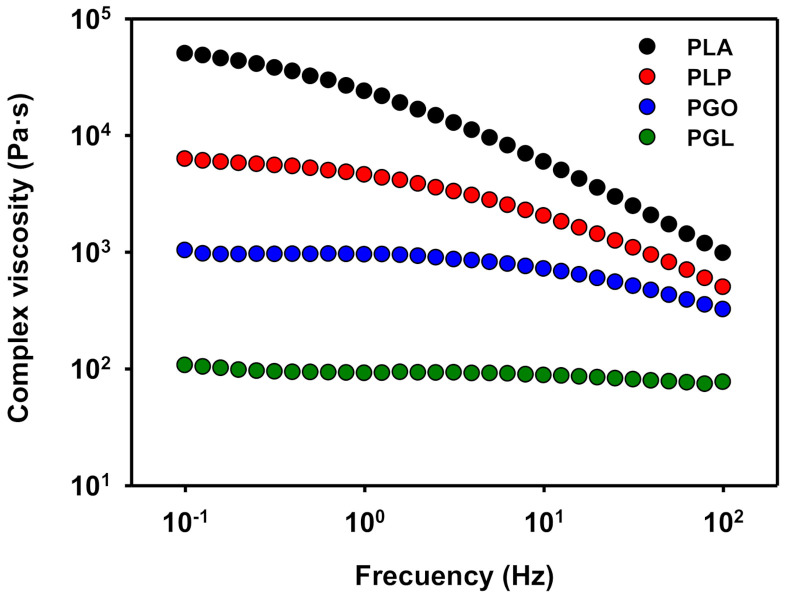
Representation of complex viscosity at 130 °C for PLA, PLP, PGO, and PGL.

When the complex viscosity is represented (Figure 10), a similar trend is found. PLA showed a shear thinning effect [66,67], which is also observed when either GO or IL are added. However, the values of viscosity of PGL were the lowest and an unexpected quasi-Newtonian behavior was observed.

## 4. Discussion

We optimized the processing conditions for pure PLA, finding that a low temperature profile is convenient for extrusion. The velocities of feeder and extruder were set constant to 10 rpm and 130 rpm, respectively. In order to decrease the extrusion temperature, an increase in pressure and torque was necessary, and higher SME values were determined. As a result, in these conditions lower temperature degradation is expected. When the composites were extruded, a decrease in the SME values was found, especially when the ionic liquid [Bmpyr]PF6 was added. Therefore, this additive improved the processability of PLA, probably because of a lubricating effect. On the other hand, we only found a drop of 17% in the SME values when GO was added.

A spectroscopic study was carried out by means of FT-IR and Raman to see if all additives were incorporated to the polymeric matrix. The presence of the ionic liquid was proved by FT-IR. The peaks at 814 and 871 cm^−1^ corresponding to the anion PF^6-^ [50,51,52,53] were perfectly seen in PLP and PGL. Furthermore, the peaks 2877 and 2964 cm^−1^ assigned to the pyrrolidinium cation were found [50,51]. However, the concentration of GO was too low for the sensitivity of the sample. Conversely, in the Raman spectra, GO was detected through the 2D band at 1585 cm^−1^ in samples PGO and PGL [68]. On the other hand, [Bmpyr]PF6 could not be observed in these spectra. In consequence, combining FT-IR and Raman, we concluded that these additives did not suffer degradation during the low temperature melting processing.

The thermogravimetric analysis of the composites showed that the addition of IL and GO increased, by approximately 20 degrees the degradation temperature of PLA. Ahmad et al. [69] proposed that reduced GO enhances the thermal degradation of PLA due to a “tortuous path” effect retarding the loose of volatile species. This mechanism is in agreement with our results. However, the thermal stability of the sample including both GO and IL decreased, and up to 11% of mass was lost at 170 °C after 8 h. Thus, the rheological tests performed at high temperatures were designed to decrease the time to a maximum of 20 min to avoid polymer degradation.

When graphene oxide was added to PLA (samples PGO and PGL), a decrease in ΔH_rel_ was observed by means of DSC. This was due to a lower crystallinity of the polymer since the carbon nanophase complicated the crystallization of PLA. On the other hand, when only IL was added a small increase in ΔH_rel_ was observed due to the lubrication between the polymer chains [70].

The determination of T_g_ was carried out by means of DSC and DMA techniques. The DSC study showed small variations of T_g_, but in the case of DMA there was a decrease for samples PLP and PGL. These disagreements can be ascribed to the fact that PLA derivatives show distorted tanδ peaks because of overlapping phenomena that occur in the glass transition region [71]. However, these lower values of T_g_ observed in the samples when [Bmpyr]PF6 was included are in agreement with the lubricating mechanism proposed according to the SME and ΔH_rel_ results.

When the viscoelastic properties are evaluated, PLA was the sample with the higher values of E’, E’’, G’, and G’’. Thus, the addition of GO, IL, or both, decreased the viscoelasticity of PLA [72]. In the glassy and rubbery states, the sample with only GO presented higher values of E’ and E’’ than PLP, in agreement with the values of SME and ΔH_rel_. Therefore, [Bmpyr]PF6 improved the ductility of PLA. However, in the melt state, the addition of GO decreased the values of the viscosity and the relaxation time more than [Bmpyr]PF6. This effect was probably due to a lower lubrication of IL since the mobility of the polymeric chains is higher when the composites are melted. However, the simultaneous addition of GO and IL provoked a decrease in the viscosity, and the melted nanocomposites presented a quasi-Newtonian behavior, and thus the processability of PLA was improved.

## 5. Conclusions

The results presented in this work highlight the effect of ionic liquid and GO on PLA-derivatives and may present a promising alternative for the development of biodegradable composites. We have prepared a series of PLA-derivatives with graphene oxide, the ionic liquid [Bmpyr]PF6, or both. All the additives are present in solid state at room temperature and the blends are simply prepared by mixing in a twin-screw extruder. The spectroscopic study (FT-IR and Raman) showed that neither GO nor [Bmpyr]PF6 presented a degradation during the extrusion. The calculations of SME and the viscoelastic behavior showed that the presence of GO and IL improved the processability of PLA, especially when both additives are included in the blend. The DSC and DMA results showed an increase in the ductility of PLA when [Bmpyr]PF6 is added due to a lubricating effect between the polymeric chains.

## Figures and Tables

**Table 1:** Technical information of graphene oxide.

Size	40 µm
Thickness	1–2 nm
Oxygen Content (XPS)	30%
BET	400 m^2^/g
Number of Layers	1–2

**Table 2:** Concentrations of additives in the PLA-based compounds.

	GO	IL
PLP	0 wt.%	5 wt.%
PGO	1 wt.%	0 wt.%
PGL	1 wt.%	5 wt.%

**Table 3:** Temperatures of extrusion profiles.

	Zone 1	Zone 2	Zone 3	Zone 4	Die
High	160 °C	185 °C	200 °C	200 °C	190 °C
Low	125 °C	145 °C	150 °C	150 °C	140 °C

**Table 4:** Processing conditions for pure PLA.

Temperature Profile	Feeder (rpm)	Extruder (rpm)	Pressure (bar)	Torque (N·m)	SME (kJ/kg)
Low	10	130	7.5	2.9	0.368
High	10	130	5	1.7	0.241

**Table 5:** Specific mechanical energy values for PLA, PLP, PGO, and PGL.

	PLA	PLP	PGO	PGL
SME (kJ/kg)	0.368	0.112	0.300	0.096

**Table 6:** FT-IR frequencies in cm^−1^ and assignments.

PLA	Assignment	IL	Assignment	GO	Assignment
2947	-C-H_3_ [46,47]	2964	C-H [50,51] CH_3_ [50,51]	3500, 3000	O-H [55,56,57]
2882	-C-H [46] -C-H_3_ [47]	2877	CH_3_ [49]	1719	C=C [56] C=O [55,56,57]
2297	CH_3_ [46,47]	1470	CH_2_ [50] CH_3_ [51,52]	1617	C=O [55,56,57] C=C [56]
1747	C=O [46,47,48,49]	871	P-F ring [50]	1037	C-OH C-O [57,58]
1452	CH_3_ [46,47,49]	814	PF6 [51,53] C-H [53]	980	COOH C-O [58]
1360	-C-H_3_ [47,49] C H-CH_3_ [46]	559	F-P-F PF6 [54]		
1180	C-O-C [47,48,49] -C-O [46]				
1130	C-O-C [47] ras CH_3_ [46]				
1090	C-O-C [46,47]				
1045	C-C [46] C-O-C [47,48,49]				
760	αCH_3_ [47] C=O [46]				
695	C=O [46]				

**Table 7:** Raman frequencies in cm^−1^ and assignments.

**PLA**	**Assignment**	**IL**	**Assignment**	**GO**	**Assignment**
711	C=O [59]	307	CH_3_-N-CH [53]	1350	D band [60]
760	C=O [59]	471	PF6 [53]	1585	G band [60]
1092	C-O-C [59]	743	PF6 [53]	2690	2D band [60]
1125	CH3 [59]	1462	CH3 [53]		
1179	C-O [59]	2984	CH3 [53]		
1386	CH3 [59]				
1450	C-H [59]				
1773	C=O [59]				

**Table 8:** Temperature of decomposition at 5, 50, and 95% weight loss.

Weight Loss (%)	PLA	PLP	PGO	PGL
5	334 °C	349 °C	334 °C	318 °C
50	365 °C	381 °C	367 °C	365 °C
95	382 °C	409 °C	383 °C	383 °C

**Table 9:** T_g_ and ΔH_rel_ for PLA, PLP, PGO, and PGL.

	PLA	PLP	PGO	PGL
T_g_	57.3 °C	55.8 °C	56.4 °C	57.7 °C
ΔH_rel_	0.51 J/g	0.53 J/g	0.18 J/g	0.14 J/g

**Table 10:** T_g_ in °C, calculated from the representations of E’, E’’, and tan δ.

	E’ (MPa)	E’’ (MPa)	tan δ
PLA	55.1 ± 0.2	54.9 ± 0.7	58.5 ± 0.2
PLP	46.1 ± 0.3	46.5 ± 0.5	55.5 ± 0.6
PGO	55.3 ± 0.2	55.8 ± 0.4	61.3 ± 0.3
PGL	42.2 ± 0.1	42.5 ± 0.6	49.5 ± 0.4

**Table 11:** Linear viscoelastic region for PLA, PLP, PGO, and PGL at different temperatures.

Temperature (°C)	PLA	PLP	PGO	PGL
110	0.01–100%	0.01–100%	0.01–100%	0.01–100%
130	0.01–100%	0.01–100%	0.01–100%	0.1–100%
150	0.01–100%	0.01–100%	0.05–100%	0.1–100%
170	0.01–100%	0.1–100%	0.1–100%	0.2–100%

## Data Availability

Data sharing not applicable.
